# Cause of death coding in Switzerland: evaluation based on a nationwide individual linkage of mortality and hospital in-patient records

**DOI:** 10.1186/s12963-019-0182-z

**Published:** 2019-03-01

**Authors:** Ueli Zellweger, Christoph Junker, Matthias Bopp, for the Swiss National Cohort Study Group, Matthias Egger, Adrian Spoerri, Marcel Zwahlen, Milo Puhan, Matthias Bopp, Martin Röösli, Michel Oris, Murielle Bochud

**Affiliations:** 1 Epidemiology, Biostatistics and Prevention Institute, University of Zurich, Hirschengraben 84, CH-8001 Zürich, Switzerland; 2 Swiss Federal Statistical Office, Espace de l’Europe 10, 2010 Neuchâtel, Switzerland

**Keywords:** Cause of death, Death certificate, Hospital discharge diagnosis, Medical record linkage, Quality monitoring, Switzerland, Validity

## Abstract

**Background:**

Cause of death statistics are an important tool for quality control of the health care system. Their reliability, however, is controversial. Comparing death certificates with their corresponding medical records is implemented only occasionally but may point to quality problems. We aimed at exploring the agreement between information in the cause of death statistics and hospital discharge diagnoses at death.

**Methods:**

Selection of disease categories was based on ICD-10 Tabulation List for Morbidity and ICD-10 Mortality Tabulation List 2. Index cases were defined as deaths having occurred among Swiss residents 2010–2012 in a hospital and successfully linked to the Swiss National Cohort. Rare, external and ill-defined causes were excluded from comparison, leaving 53,605 deaths from vital statistics and 47,311 deaths from hospital discharge statistics. For 95% of individuals, respective information from the 2000 census could be retrieved and used for multiple logistic regression.

**Results:**

For 83% of individuals the underlying cause of death could be traced among hospital diagnoses and for 77% the principal hospital diagnosis among the cause of death information. Mirroring different evaluation of complex situations by individual physicians, rates of agreement varied widely depending on disease/cause of death, but were generally in line with similar studies. Multiple logistic regression revealed however significant variation in reporting that could not entirely be explained by age or cause of death of the deceased suggesting differential exploitation of available diagnosis information.

**Conclusion:**

Substantial regional variation and lower agreement rates among socially disadvantaged groups like single, less educated, or culturally less integrated persons suggest potential for improving reporting of diagnoses and causes of death by physicians in Switzerland. Studies of this kind should be regularly conducted as a quality monitoring.

## Introduction

Cause of death statistics are an important tool for monitoring the health of populations and for responding effectively to changing epidemiological circumstances [1, 2]. They are also a tool for quality control of the health care system. For example deaths due to causes that should not be fatal in the presence of effective medical care, known as amenable mortality, is an indicator of national levels of personal health-care access and quality [3]. Because of the long-standing supervision by WHO the statistics are, in principle, comparable over time and between countries [4]. Their reliability, however, is controversial [5–7]. Substantial variation in certification practices between countries is a known problem [8, 9]. Even within the same country, “different coding practices, socio-cultural milieus, and individual socio-demographic characteristics such as age are most likely to influence the cause of death assigned on the death certificate” [10].

For these reasons it is important to evaluate the reliability of cause-of-death assignment and coding. Usually validation consists in obtaining the coding of the same set of death certificates from different raters [1, 7, 10, 11]. Unfortunately an agreement between the original and the reviewed data may us tell more about reproducibility and less aboutaccuracy of the information [12]. The forms look correct but do not provide an accurate description of the case [13]: Less frequently, death certificates were compared with their corresponding medical records (e.g., [14, 15]), a procedure rated as too slow and expensive for routine use [13] and therefore implemented only occasionally and studies spanning short periods [15–17]. One of these studies, however, confirmed that medical incompatibility between underlying cause of death and main hospital discharge condition is a marker for greater risk of erroneous cause-of-death certification [16].

Switzerland introduced cause of death statistics in 1876. Certifying by a physician became mandatory a few years later [18]. In the assessments by WHO, the quality of Swiss data was rated as medium [19] or medium-high [5], due to a high proportion of ill-defined causes. This is in line with a recent evaluation by the Global Burden of Disease 2016 Causes of Death Collaborators, which concluded that in Switzerland since 2000 a larger proportion of “well certified deaths” than in neighboring Germany and France (but still not reaching the proportions in neighboring Austria and Italy) [20]. The only evaluation studies comparing cause of death statistics with other medical information date from the 1980s [21, 22]. More recent validation studies were restricted to death certificates and the implications of the adoption of ICD-10 in 1995, trying to identify correction factors with which to correct time series [23, 24].

In Swiss hospitals, cause of death reporting to the Federal Statistical Office is generally in the charge of the responsible attending physician, who also completes the medical files which in turn serve as the basis for the standardized registration of diseases and treatments in the hospital discharge statistics. It is therefore compelling to analyze the consistency of ICD mapping on death certificates and in hospital discharge statistics. Agreement of both, however, is still more a marker for reproducibility than validity of the information. Nevertheless, incompatibility of diagnoses and causes of death may point to quality problems [14].

In contrast to others we did not target an overall quality measure for hospital and cause of death statistics in Switzerland (e.g., the proportion of ill-defined causes) but aimed at assessing the reliability of as many as possible specified diagnoses / causes of death. Within this frame, we first aim at exploring to what extent the underlying cause of death in the cause of death statistics is in agreement with principal and additional diagnoses registered at hospital discharge in the Medical Statistics of Swiss Hospitals (MS). Second, we aim at exploring the opposite, i.e., the agreement of principal diagnosis at hospital discharge with cause of death information on the death certificate. Which causes of death can be traced in the MS especially well or badly and which principal hospital diagnoses can be traced in the cause of death statistics especially well or poorly? In addition, it may be of interest to know which diseases have the most similarity or variation of agreement rates between the two approaches. Finally, we aimed at evaluating combined vs. separate disease categories where the related ICD-codes may not always be easily distinguishable, and at looking for sociodemographic determinants that could influence the agreement between hospital and cause of death data.

For all analyses comparing hospital discharge diagnoses with cause of death information, one has, however, to be aware that the definitions of principal hospital diagnosis and underlying cause of death do not necessarily agree. Cause of death statistics have to deliver a unicausal result, also for multimorbid subjects for whom this concept is not appropriate. On the other hand, medical coding in hospitals first of all serves reimbursement and this may result in distortions [12]. The principal hospital diagnosis may also be a complication of the underlying cause of death. For these reasons, taking into account additional diagnoses as well as concomitant causes of death will be essential.

## Methods

### Data

#### Cause of death statistics

In Switzerland, death has to be certified by a physician, who can report the immediate and the underlying cause of death as well as up to two secondary causes, generally referring to concomitant diseases. The underlying cause of death is defined as a) “the disease or injury which initiated the train of events leading directly to death” or (b) “the circumstances of the accident or violence which produced the fatal injury” [25].

After possible inquiries with the certifying physician, the Swiss Federal Statistical Office centrally codes all cause of death information according to the International Statistical Classification of Diseases and Related Health Problems (ICD-10) and assigns a primary, so-called “definitive” cause of death, which – being in most cases identical with the underlying cause on the death certificate – is decisive for all official publications.

For those who died between 55 and 94 years of age, the probability that more than one cause of death is reported as well as the average number of reported causes/diseases gradually increase and only slightly decrease thereafter. In the average, those deceased in a hospital get more diagnoses than those deceased in a long-term care facility, while those deceased at other places (mostly private home) get the fewest diagnoses listed. Restricted to natural deaths in the age span between 60 and 84, men in the average get slightly more diagnoses than women of the same age.

#### Medical statistics of Swiss hospitals (MS)

Since 1998 all hospitals in Switzerland have to report their inpatient stays to the Swiss Federal Statistical Office [26]. For every hospital discharge, one principal diagnosis (defined as main reason for medical service or disease having caused most expenditure during this hospital stay) and up to 49 additional diagnoses (previously known or detected during hospitalization) were coded according to the International Statistical Classification of Diseases and Related Health Problems (ICD-10).

In contrast to cause of death statistics, ICD coding as well as an anonymous encryption of the personal identifier is conducted on-site by the hospitals, precluding further inquiries by the Federal Statistical Office [27].

Though this procedure is fully anonymized, hospitalizations of the same individual can be aggregated. Sociodemographic information in the MS is limited to gender, age class and region of residence and there is no established link to the cause of death statistics. Only for decedents, hospitals are obliged to report full date of birth. Using full date of birth, sex, and a geographical identifyer, an anonymous record linkage with the Swiss National Cohort [28] could be successfully established [29], providing additional socio-demographic information (educational level, place of birth, principal language, household type) from the 2000 census for 95% of deceased individuals in the study population.

### Disease categories

In hospitals, external causes are generally coded within the chapter “Injury, poisoning and certain other consequences of external causes” (S00-T98) rather than the chapter “External causes of morbidity and mortality” (X00-Y99) which is relevant to mortality statistics. In these cases, as well as in the chapter “Symptoms, signs and abnormal clinical and laboratory findings” (R00-R99), an agreement between hospital discharge and mortality statistics a priori cannot be expected and we therefore did not consider external causes.

Selection of categories for analysis and the tables in the results section was based on

1. ICD-10 Tabulation List for Morbidity [25] (A00-Q99: 266 of originally 298 items)
2. ICD-10 Mortality Tabulation List 2: General mortality - Selected list [25] (A00-Q99: 69 of originally 80 items)

For the selection of categories we eliminated duplicates and excluded nonspecific (“other...”) categories and those with fewer than 30 deaths in 2010–2012 (in both, “definitive” cause of death^1^ and principal hospital discharge diagnosis^2^).

Generally, the list for morbidity is more specific than the selected list for mortality. However there are two exceptions (Malignant neoplasm of ovary, C56; Multiple myeloma and malignant plasma cell neoplasms, C90) which were preferred over the corresponding categories in the list for morbidity. Dementia (F01–03) and Alzheimer’s disease (G30) were combined into one category, since in every day practice this differentiation is not reliable [30]. Similarly, we put emphasis on keeping the summary as well as the detailed categories for diseases with internationally well-known differentiation problems, i.e. malignancies of the colorectum (C18-C21), coronary heart disease (I20-I25), cerebrovascular diseases (I60-I69) and liver disease (K70-K76). Consequently, we added a respective summary category for malignant uterine tumors (C53-C55). After these modifications, 76 disease master categories (64 from the Tabulation List for Morbidity, 10 from the Mortality Tabulation List, and the two extra items “dementia/Alzheimer’s disease” and “all malignant uterine tumors”) remained for more thorough analysis.

### Statistical analysis

First, we assessed among those deceased in a hospital to what extent the “definitive” cause of death assigned by the Swiss Federal Statistical Office was in agreement with a) the principal, and b) any hospital discharge diagnosis.

Second, we assessed among the same study population to what extent the principal hospital discharge diagnosis was in agreement with underlying, immediate and contributory causes of death recorded.

SPSS 25 (IBM Corp, 2017) was used to calculate proportions of decedents who had a) the “definitive” underlying cause of death also as the principal or an additional discharge diagnosis for their terminal hospital stay, and b) who had the principal hospital discharge diagnosis also registered as the underlying or a contributory cause of death in the mortality statistics. Agreement between hospital diagnoses and causes of death was measured by kappa coefficients using SPSS 25. Logistic regression was performed using STATA 13.1 (StataCorp, 2013) to assess the influence of socio-demographic variables on the agreement of hospital diagnoses and the underlying cause of death.

## Results

### Study population

**Fig. 1:**
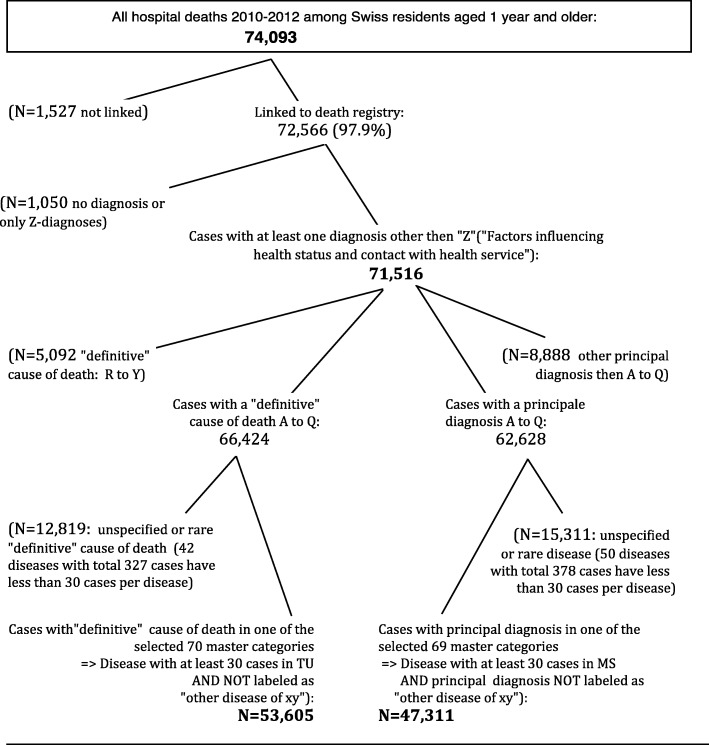
Derivation of the study population. Hospital deaths 2010–2012 among Swiss residents aged 1 year and older, which could be linked to the death registry and categorized into one of 70 master categories of cause of death or into one of 69 master categories of principal hospital discharge diagnosis

Index cases were defined as deaths having occurred among Swiss residents 2010–2012 in a hospital and successfully linked to a cause of death statistics record. Starting with 74,093 deaths registered in the hospital discharge statistics, 72,566 were found to be index cases (97.9%) (Fig. 1). Among those, 1050 cases had no or exclusively “Z”-diagnoses (“Factors influencing health status and contact with health services”) and were excluded from analyses. The final number of valid cases is therefore 71,516.

Excluding external and ill-defined causes, tracing causes of death in the hospital discharge statistics was restricted to those with a “definitive” cause of death in the range A00-Q99 (*N* = 66,424), and tracing diagnoses in the cause of death statistics to those with a principal hospital discharge diagnosis in the range A00-Q99 (*N* = 62,628).

Overall, 5.3% (men) and 5.8% (women) had only one – i.e., the principal – diagnosis. On average, 8.1 (men) and 7.6 (women) diagnoses were reported, with a maximum of almost 11 among boys aged 10–14 years and a minimum of 5.8 among women aged 95 years and more.

With focus on tracing cause of death in hospital diagnoses, analyses encompassed 53,605 deaths in 70 master categories, whereas for tracing the principal diagnosis in cause of death information, there were 47,311 deaths in 69 master categories (with 64 of these master categories being identical – see footnote above). From the overall 76 disease master categories, 9 were divided into overall 21 subcategories, inducing a final tabulation set of 97 disease categories.

### “Definitive” underlying cause of death found in diagnoses of the terminal hospital discharge record

Among the 70 selected master categories, for 83% (44,740/53,605) of cases the individual “definitive” underlying cause of death could be found among the principal or additional diagnoses reported for the terminal hospital stay of the same individual.

For most categories, in at least 70% of cases the underlying cause of death was also reported in the Medical Statistics of the Swiss Hospitals (Table 1). For cancer categories this proportion was generally over 80%. The largest agreements were observed for diseases of the liver (93%), multiple myeloma, lung and brain cancer (all 92%), diseases of the pancreas (91%), renal failure, breast and pancreatic cancer (all 90%), and cerebrovascular diseases (89%).

**Table 1:** Agreement of underlying cause of death with diagnoses registered for the terminal hospitalization

“Definitive” cause of death found as ...		Principal diagnosis	In any diagnosis		
Disease Category	N	%	N	%	Kappa
Chapter 1: A00-B99 Certain infectious and parasitic diseases					
Diarrhoea / gastro-enteritis of presumed infectious origin	109	17%	41	38%	0.12
Septicaemia	503	63%	383	76%	0.08
Human immunodeficiency virus [HIV] disease	84	31%	70	83%	0.60
Mycoses	30	47%	26	87%	0.02
Chapter 2: C00-D49 Neoplasms					
Malignant neoplasm of lip, oral cavity / pharynx	727	59%	607	83%	0.82
Malignant neoplasm of oesophagus	825	57%	726	88%	0.86
Malignant neoplasm of stomach	910	64%	793	87%	0.85
Malignant neoplasm of colon, rectum / anus	2917	58%	2578	88%	0.87
Malignant neoplasm of colon	1939	55%	1607	83%	0.83
Malignant neoplasm of rectosigmoid junction, rectum, anus / anal canal	978	53%	809	83%	0.79
Malignant neoplasm of liver / intrahepatic bile ducts	1252	57%	1025	82%	0.81
Malignant neoplasm of pancreas	2019	68%	1826	90%	0.91
Malignant neoplasm of larynx	137	51%	103	75%	0.71
Malignant neoplasm of trachea, bronchus / lung	5825	62%	5359	92%	0.91
Malignant neoplasm of bone / articular cartilage	59	46%	40	68%	0.54
Malignant melanoma of skin	505	44%	402	80%	0.85
Malignant neoplasms of mesothelial / soft tissue	599	55%	469	78%	0.76
Malignant neoplasm of breast	2226	51%	2013	90%	0.88
Malignant neoplasm of cervix uteri / uterus	470	55%	392	83%	0.83
Malignant neoplasm of cervix uteri	133	57%	107	80%	0.81
Malignant neoplasm of other / unspecified parts of uterus	337	51%	271	80%	0.80
Malignant neoplasm of ovary	766	54%	658	86%	0.88
Malignant neoplasm of prostate	1751	47%	1559	89%	0.79
Malignant neoplasm of bladder	873	50%	700	80%	0.75
Malignant neoplasm of eye / adnexa	48	40%	35	73%	0.74
Malignant neoplasm of brain	671	75%	615	92%	0.90
Hodgkin’s disease	60	43%	44	73%	0.66
Non-Hodgkin’s lymphoma	934	56%	799	86%	0.81
Multiple myeloma / malignant plasma cell neoplasms	555	58%	513	92%	0.86
Leukaemia	1116	56%	961	86%	0.80
Chapter 3:D50-D89 Diseases of the blood and blood-forming organs / certain disorders involving the immune mechanism					
Anaemias	136	36%	104	76%	0.01
Haemorrhagic conditions / other diseases of blood (−forming organs)	74	35%	59	80%	0.02
Chapter 4: E00-E89 Endocrine, nutritional and metabolic diseases					
Diabetes mellitus	657	13%	511	78%	0.08
Obesity	61	16%	42	69%	0.06
Chapter 5: F01-F99 Mental, Behavioral and Neurodevelopmental disorders					
Mental / behavioural disorders due to psychoactive substance use	121	11%	93	77%	0.05
Mental / behavioural disorders due to use of alcohol	85	15%	66	78%	0.07
Mental / behavioural disorders due to other psychoactive substance use	36	0%	25	69%	0.03
Schizophrenia, schizotypal / delusional disorders	41	56%	34	83%	0.14
Chapter 6: G00-G99 Diseases of the nervous system					
Inflammatory diseases of the central nervous system	71	49%	57	80%	0.27
Parkinson’s disease	300	19%	224	75%	0.37
Dementia / Alzheimer’s disease	862	29%	628	73%	0.22
Dementia	587	13%	394	67%	0.14
Alzheimer’s disease	275	36%	185	67%	0.33
Multiple sclerosis	76	14%	65	86%	0.60
Epilepsy	114	48%	88	77%	0.07
Cerebral palsy / other paralytic syndromes	66	11%	49	74%	0.02
Chapter 9: I00-I99 Diseases of the circulatory system					
Chronic rheumatic heart disease	126	12%	45	36%	0.05
Hypertensive diseases	1606	5%	1115	69%	0.05
Essential (primary) hypertension	105	0%	42	40%	0.00
Other hypertensive diseases	1501	6%	872	58%	0.13
Ischaemic heart diseases	6746	40%	5120	76%	0.44
Acute myocardial infarction	2950	63%	2300	78%	0.59
Other ischaemic heart diseases	3796	7%	2314	61%	0.25
Pulmonary embolism	464	50%	325	70%	0.25
Conduction disorders / cardiac arrhythmias	1204	24%	867	72%	0.06
Heart failure	1046	52%	827	79%	0.09
Cerebrovascular diseases	4622	77%	4099	89%	0.63
Intracranial haemorrhage	1774	81%	1526	86%	0.64
Cerebral infarction	1171	70%	928	79%	0.43
Stroke, not specified as haemorrhage or infarction	1320	18%	294	22%	0.29
Other cerebrovascular diseases	357	6%	111	31%	0.08
Atherosclerosis	263	49%	201	76%	0.14
Arterial embolism / thrombosis	180	52%	120	67%	0.26
Phlebitis, thrombophlebitis, venous embolism / thrombosis	89	17%	48	54%	0.06
Chapter 10: J00-J99 Diseases of the respiratory system					
Pneumonia	1845	36%	1471	80%	0.18
Chronic lower respiratory diseases	2188	32%	1833	84%	0.36
Bronchitis, emphysema / other chronic obstructive pulmonary diseases	2119	32%	1772	84%	0.37
Asthma	51	24%	25	49%	0.10
Bronchiectasis	18	11%	12	67%	0.20
Chapter 11: K00-K95 Diseases of the digestive system					
Gastric / duodenal ulcer	275	64%	220	80%	0.34
Diseases of appendix	36	64%	29	81%	0.54
Inguinal hernia	38	58%	27	71%	0.27
Crohn’s disease / ulcerative colitis	56	25%	37	66%	0.30
Paralytic ileus / intestinal obstruction without hernia	620	62%	532	86%	0.23
Diverticular disease of intestine	455	57%	368	81%	0.49
Diseases of the liver	1367	54%	1269	93%	0.36
Alcoholic liver disease	1009	44%	808	80%	0.59
Other diseases of liver	358	43%	303	85%	0.12
Cholelithiasis / cholecystitis	249	45%	216	87%	0.40
Acute pancreatitis / other diseases of the pancreas	211	60%	192	91%	0.47
Chapter 12: L00-L99 Diseases of the skin and subcutaneous tissue					
Infections of the skin / subcutaneous tissue	70	23%	40	57%	0.13
Chapter 13: M00-M99 Diseases of the musculoskeletal system / connective tissue					
Rheumatoid arthritis / other inflammatory polyarthropathies	66	17%	52	79%	0.10
Arthrosis	114	27%	41	36%	0.10
Systemic connective tissue disorders	139	29%	100	72%	0.26
Soft tissue disorders	55	36%	41	75%	0.06
Disorders of bone density / structure	35	34%	20	57%	0.02
Osteomyelitis	32	16%	22	69%	0.15
Chapter 14: N00-N99 Diseases of the genitourinary system					
Renal tubulo-interstitial diseases	70	20%	48	69%	0.09
Renal failure	687	40%	617	90%	0.04
Hyperplasia of prostate	35	11%	17	49%	0.03
Chapter 17: Q00-Q99 Congenital malformations, deformations and chromosomal abnormalities					
Congenital malformations of the circulatory system	66	11%	32	48%	0.26
Total Cases (sum of 70 master categories)	53,605	50%	44,740	83%	

Low agreement was observed for not specified stroke (22%) and other cerebrovascular diseases (31%), arthrosis and chronic rheumatic heart disease (both 36%), diarrhoea/gastro-enteritis (38%), and primary hypertension (40%).

Using the kappa coefficient, the variation between cancer categories (kappa generally > 0.70, the highest values were obtained for pancreatic cancer, kappa = 0.91, and brain cancer, kappa = 0.90) and the other diseases (kappa generally < 0.30) became even more apparent: notable exceptions were only cerebrovascular diseases (kappa = 0.63), multiple sclerosis (kappa = 0.60), AMI and alcoholic liver disease (both kappa = 0.59).

Based on the proportion of agreement the following master categories substantially outperformed the respective subcategories: cerebrovascular diseases, hypertensive diseases, liver disease and Alzheimer’s disease/other dementia. Also for colorectal and uterine cancer the master category performed better than the subcategories, whereas for chronic lower respiratory diseases vs. COPD, ischaemic heart diseases vs. AMI and mental disorders due to substance abuse this was not the case. Based on the kappa coefficient, only the colorectal and uterine cancer master categories had an unambiguous advantage over the respective subcategories.

Restricted to the principal hospital discharge diagnosis, agreement with the “definitive” underlying cause of death decreased to 50%, with most distinctive decreases for multiple sclerosis (found in 86% of linked hospital discharge records, but only in 14% as the principal diagnosis), diabetes mellitus (78 -- > 13%), hypertensive diseases (69 -- > 5%), mental disorders due to alcohol use (78 -- > 15%), cerebral palsy (74 -- > 11%), Parkinson’s disease (75 -- > 19%), Alzheimer’s disease/other dementia (73 -- > 19%), HIV disease (83 -- > 31%), chronic lower respiratory diseases (84 -- > 32%), and renal failure (90 -- > 40%).

### Principal hospital discharge diagnosis found in cause of death statistics

Among the 69 selected master categories, for 77% (36,456/47,311) of cases the principal diagnosis at the terminal hospital stay could be traced among the cause of death information for the respective individual. Rates of agreement were highest for cancers (with up to 98% for breast cancer, 97% for prostate cancer and multiple myeloma, 96% for melanoma of skin and lung cancer, 93% for cerebrovascular diseases, 92% for multiple sclerosis and ischaemic heart diseases, and 90% for chronic lower respiratory disease and liver disease (Table 2).

**Table 2:** Agreement of principal hospital discharge diagnosis with cause of death statistics

Principal diagnosis found as ...		“Definitive” cause of death	In any cause of death		
Disease Category	N	%	N	%	Kappa
Chapter 1: A00-B99 Certain infectious and parasitic diseases					
Diarrhoea / gastro-enteritis of presumed infectious origin	90	20%	31	34%	0.18
Septicaemia	4330	7%	2108	49%	0.41
Human immunodeficiency virus [HIV] disease	30	87%	27	90%	0.41
Mycoses	95	15%	45	47%	0.25
Chapter 2: C00-D49 Neoplasms					
Malignant neoplasm of lip, oral cavity / pharynx	462	92%	434	94%	0.65
Malignant neoplasm of oesophagus	524	90%	483	92%	0.68
Malignant neoplasm of stomach	657	88%	587	89%	0.71
Malignant neoplasm of colon, rectum / anus	1820	93%	1722	95%	0.67
Malignant neoplasm of colon	1187	90%	1084	91%	0.64
Malignant neoplasm of rectosigmoid junction, rectum, anus / anal canal	634	82%	534	84%	0.61
Malignant neoplasm of liver / intrahepatic bile ducts	820	87%	726	89%	0.67
Malignant neoplasm of pancreas	1474	93%	1384	94%	0.77
Malignant neoplasm of larynx	85	82%	71	84%	0.49
Malignant neoplasm of trachea, bronchus / lung	3793	95%	3644	96%	0.72
Malignant neoplasm of bone / articular cartilage	46	59%	27	59%	0.49
Malignant melanoma of skin	235	95%	225	96%	0.56
Malignant neoplasms of mesothelial / soft tissue	387	84%	330	85%	0.64
Malignant neoplasm of breast	1165	97%	1143	98%	0.59
Malignant neoplasm of cervix uteri / uterus	289	89%	261	90%	0.63
Malignant neoplasm of cervix uteri	85	89%	76	89%	0.66
Malignant neoplasm of other / unspecified parts of uterus	204	85%	177	87%	0.59
Malignant neoplasm of ovary	444	93%	421	95%	0.66
Malignant neoplasm of prostate	879	94%	849	97%	0.47
Malignant neoplasm of bladder	529	83%	455	86%	0.56
Malignant neoplasm of brain	543	93%	504	93%	0.82
Hodgkin’s disease	35	74%	26	74%	0.43
Non-Hodgkin’s lymphoma	589	89%	539	92%	0.62
Multiple myeloma / malignant plasma cell neoplasms	337	95%	327	97%	0.65
Leukaemia	710	88%	650	92%	0.62
Chapter 3:D50-D89 Diseases of the blood and blood-forming organs / certain disorders involving the immune mechanism					
Anaemias	202	24%	101	50%	0.08
Haemorrhagic conditions / other diseases of blood (−forming organs)	279	9%	72	26%	0.14
Chapter 4: E00-E89 Endocrine, nutritional and metabolic diseases					
Diabetes mellitus	180	46%	132	73%	0.04
Malnutrition	60	10%	20	33%	0.07
Volume depletion	113	4%	28	25%	0.08
Chapter 5: F01-F99 Mental, Behavioral and Neurodevelopmental disorders					
Mental / behavioural disorders due to psychoactive substance use	65	20%	31	48%	0.04
Mental / behavioural disorders due to use of alcohol	45	29%	21	47%	0.05
Mental / behavioural disorders due to other psychoactive substance use	20	0%	9	45%	0.03
Schizophrenia, schizotypal / delusional disorders	122	19%	68	56%	0.36
Mood [affective] disorders	180	7%	93	52%	0.22
Neurotic, stress-related and somatoform disorders	55	2%	5	9%	0.05
Chapter 6: G00-G99 Diseases of the nervous system					
Inflammatory diseases of the central nervous system	88	40%	57	65%	0.46
Parkinson’s disease	73	78%	66	90%	0.15
Dementia / Alzheimer’s disease	465	54%	365	78%	0.20
Dementia	229	34%	146	64%	0.10
Alzheimer’s disease	236	42%	118	50%	0.28
Epilepsy	296	19%	159	54%	0.30
Transient cerebral ischaemic attacks and related syndromes	34	0%	3	9%	0.07
Cerebral palsy / other paralytic syndromes	78	9%	20	26%	0.09
Chapter 9: I00-I99 Diseases of the circulatory system					
Chronic rheumatic heart disease	88	17%	17	19%	0.10
Hypertensive diseases	272	31%	174	64%	0.02
Essential (primary) hypertension	6	0%	2	33%	0.00
Other hypertensive diseases	266	32%	161	61%	0.03
Ischaemic heart diseases	3144	85%	2890	92%	0.30
Acute myocardial infarction	2692	68%	1980	74%	0.62
Other ischaemic heart diseases	452	61%	365	81%	0.05
Pulmonary embolism	648	36%	521	80%	0.34
Conduction disorders / cardiac arrhythmias	1864	16%	1087	58%	0.13
Heart failure	3842	14%	2043	53%	0.39
Cerebrovascular diseases	4829	74%	4476	93%	0.69
Intracranial haemorrhage	2181	66%	1929	88%	0.76
Cerebral infarction	2081	40%	991	48%	0.52
Stroke, not specified as haemorrhage or infarction	460	53%	312	68%	0.23
Other cerebrovascular diseases	141	16%	54	38%	0.05
Atherosclerosis	448	29%	276	62%	0.22
Arterial embolism / thrombosis	282	33%	136	48%	0.41
Phlebitis, thrombophlebitis, venous embolism / thrombosis	70	21%	35	50%	0.08
Chapter 10: J00-J99 Diseases of the respiratory system					
Pneumonia	2926	22%	2027	69%	0.32
Chronic lower respiratory diseases	892	78%	801	90%	0.20
Bronchitis, emphysema / other chronic obstructive pulmonary diseases	868	78%	773	89%	0.21
Asthma	20	60%	13	65%	0.07
Bronchiectasis	5	40%	2	40%	0.10
Chapter 11: K00-K95 Diseases of the digestive system					
Gastric / duodenal ulcer	376	47%	216	57%	0.51
Gastritis and duodenitis	31	16%	9	29%	0.15
Diseases of appendix	33	70%	24	73%	0.62
Inguinal hernia	56	39%	27	48%	0.45
Paralytic ileus / intestinal obstruction without hernia	1092	35%	746	68%	0.45
Diverticular disease of intestine	429	60%	300	70%	0.53
Diseases of the liver	1161	64%	1040	90%	0.40
Alcoholic liver disease	560	79%	477	85%	0.49
Other diseases of liver	612	25%	406	66%	0.23
Cholelithiasis / cholecystitis	199	56%	130	65%	0.44
Acute pancreatitis / other diseases of the pancreas	179	71%	158	88%	0.54
Chapter 12: L00-L99 Diseases of the skin and subcutaneous tissue					
Infections of the skin / subcutaneous tissue	71	23%	23	32%	0.19
Chapter 13: M00-M99 Diseases of the musculoskeletal system / connective tissue					
Arthrosis	112	28%	47	42%	0.21
Systemic connective tissue disorders	65	63%	50	77%	0.21
Soft tissue disorders	122	16%	37	30%	0.19
Disorders of bone density / structure	94	13%	33	35%	0.08
Osteomyelitis	35	14%	18	51%	0.29
Chapter 14: N00-N99 Diseases of the genitourinary system					
Renal tubulo-interstitial diseases	87	16%	36	41%	0.20
Renal failure	1206	23%	826	68%	0.13
Total Cases (sum of 69 master categories)	47,311	57%	36,442	77%	

Again, using the kappa coefficient, the variation between cancer categories (kappa generally ≥0.59, maxima in brain cancer, kappa = 0.82, and pancreatic cancer, kappa = 0.77) and the other diseases (kappa generally< 0.30) is obvious, however less distinct than in the first analysis. Notable exceptions from the generally low kappa values were only cerebrovascular diseases (kappa = 0.69) and AMI (kappa = 0.62).

As to the proportion of agreement, the master categories cerebrovascular diseases, ischaemic heart diseases and Alzheimer’s disease/other dementia substantially outperformed the respective subcategories, but also in liver disease and colorectal cancer the master categories performed clearly better than the average of the respective subcategories. Based on the kappa coefficient the advantage of the master category was limited to colorectal cancer.

Restricted to the “definitive” underlying cause of death, agreement with principal hospital discharge diagnosis decreased to 57%, with most distinctive decreases for pneumonia (69 -- > 22%), renal failure (68 -- > 23%), pulmonary embolism (80 -- > 36%), septicaemia (49 -- > 7%), cardiac arrhythmias (58 -- > 16%) and heart failure (53 -- > 14%).

Agreement between principal hospital discharge diagnosis and the original underlying cause of death was even slightly lower (54%; 25,762/47,311), with some exceptions from this general pattern (most notably atherosclerosis, hypertensive diseases, pneumonia, liver disease and mental disorders due to alcohol use).

### Determinants of agreement between hospital and death records

**Fig. 2:**
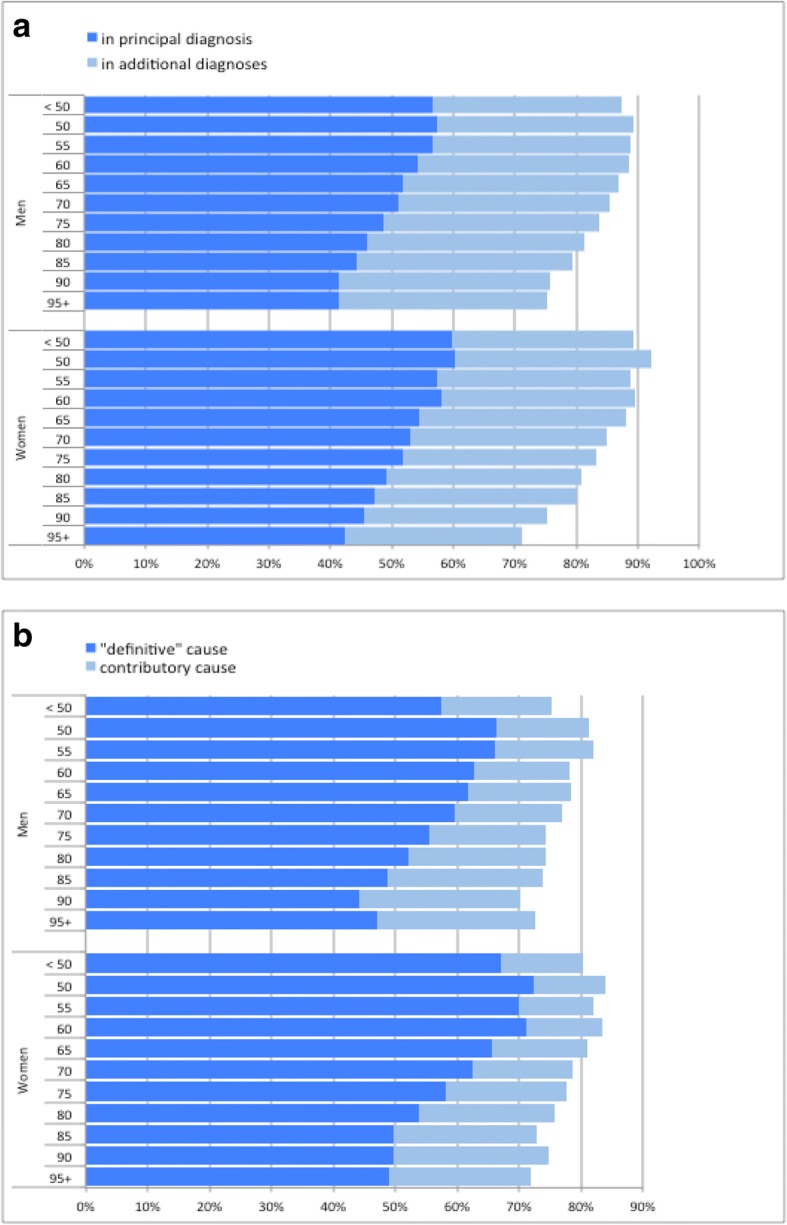
Agreement of causes of death and hospital discharge diagnoses. **a** Agreement of underlying cause of death with diagnoses (principal or additional) registered for the terminal hospitalization, by sex and age (*N* = 53,605). **b** Agreement of principal hospital discharge diagnosis at death with “definitive” and contributory causes of death, by sex and age (*N* = 47,339)

For men and even more for women deceased after age 60, agreement of the “definitive” cause of death with any hospital diagnosis decreased with increasing age from around 90% to around 75% among those aged 90 years and more (Fig. 2a). This decrease is almost entirely due to decreasing agreement with principal hospital discharge diagnosis, while agreement with additional hospital diagnoses varied only slightly (between 29 and 35%).

The agreement of principal hospital diagnosis with cause of death information decreases with increasing age, too (Fig. 2b). Proportions of agreement however are lower and the general decrease applies to both, “definitive” and additional causes of death.

### Socio-demographic determinants for individual level disagreement

For 50,995 of the 53,605 deceased persons with a “definitive” cause of death classified in one of the 70 master categories (95.1%), socio-demographic information from the 2000 census could be retrieved. Multiple logistic regression revealed substantial impact of socio-demographic determinants on agreement of “definitive” cause of death and any hospital discharge diagnoses. Compared to the Lake Geneva region, the only predominantly French-speaking region, agreement at the individual level was higher in all parts of Switzerland, most obviously in Zurich (OR = 1.28; 95% CI 1.15–1.41) and Espace Mittelland (OR = 1.21; 1.11–1.31) (Table 3). Higher agreement also applied to ever married individuals and those with a higher educational level, whereas French speaking individuals and even more so those speaking a non-European language had significantly lower odds of agreement than their German speaking counterparts. As in the descriptive analysis, age but not sex of the deceased had substantial impact on the odds of agreement.

**Table 3:** Agreement of underlying cause of death with diagnoses (principal or additional) registered for the terminal hospitalization: multiple logistic regression (*N* = 50,995)

	“Definitive” cause of death found in principal or additional hospital discharge diagnoses		
	OR	95% CI	*P*-value
Major region			
Lake Geneva Region	1 (ref.)		
Zurich	1.28	1.15–1.41	< 0.001
Espace Mittelland	1.21	1.11–1.31	< 0.001
Central	1.13	1.01–1.28	0.04
Northwest	1.10	.999–1.21	0.05
Eastern	1.11	.997–1.23	0.06
Ticino	1.15	.992–1.34	0.06
Marital status of deceased (from death certificate)			
Single	1 (ref.)		
Married	1.28	1.18–1.39	< 0.001
Widowed	1.18	1.08–1.29	< 0.001
Divorced	1.21	1.09–1.35	< 0.001
Principal language of deceased (from 2000 census)			
German	1 (ref.)		
French	.90	.83–.98	0.01
Italian	1.002	.89–1.12	0.97
Other European	.91	.77–1.07	0.26
Other	.73	.59–.90	< 0.01
Educational level of deceased (from 2000 census)			
Mandatory schooling	1 (ref.)		
Upper secondary	1.06	1.002–1.11	0.04
Tertiary	1.08	1.002–1.17	0.04
Age of deceased (year)	.976	.973–.978	< 0.001

Focussing on agreement of “definitive” cause of death and principal hospital discharge diagnosis provided similar however attenuated patterns, with highest agreement in Northwestern Switzerland but no significant variation by language category.

For 95.1% (44,993/47,311) of deceased individuals with a principal diagnosis in one of the 69 master categories, information from the 2000 census could be retrieved. In multiple logistic regression, retrieval in cause of death information were again significantly lower among single and less educated individuals and higher in Northwestern Switzerland, but not among native-speakers of non-Swiss languages (data not shown).

## Discussion

In Switzerland, on an individual level cause of death information in official cause of death statistics and diagnoses in the hospital discharge statistics are generally compatible, especially if additional diagnoses and contributory causes are taken into account. In 83% of analyzed deaths, the underlying cause of death could be traced in one of the diagnoses of the terminal hospital discharge and in 77% the principal discharge diagnosis was also reported as primary or contributory cause of death (principal diagnosis = primary cause: 57%). This is fairly in line with Johansson&Westerling who found agreement of principal hospital condition with underlying cause of death of 59% and with any information on the death certificate of 83% [13]. The latter number is somewhat lower than the 89% reported in two studies based on automated coding of diseases and causes of death [14, 17] reducing variation in the evaluation of complex situations but not necessarily resulting in higher validity of agreement [12].

The proportion of agreement varied widely depending on disease/cause of death, but was generally in line with known patterns from similar studies [13, 15, 22] as well as from intercoder agreement studies (e.g., [1, 11]), i.e., excellent in cancers and cerebrovascular disease and very good in respiratory and liver diseases. Discordance in less clearly defined chronic diseases may therefore be due to a large part to increased difficulty in reaching a consensus on diagnosis.

Of note, for individuals with long-lasting chronic diseases like multiple sclerosis, the immediate reason for a hospitalization is often due rather to sequelae which consequently appear as the principal hospital diagnosis. As studies from Sweden suggest, a substantial part of individual cases with discrepancies may be attributable to incomplete or inadequate transformation of the diagnostic information in the patient charts into disease assignment [31] or mistakes in death certification [16]. The fact that the logic and structure of the ICD differs from the clinical way of thinking [12] may also play a role, as well as the well-known difficulty to define a single, disease-specific, underlying cause of death in older people [6].

The proportion of agreement that was found between the underlying cause of death and hospital diagnoses was very similar to that found by Minder&Zingg in their 1979 sample of deaths in Switzerland [22]. The only notable differences were somewhat higher agreement rates in our study for malignant neoplasms of colon, rectum and larynx, while for leukaemia and diabetes mellitus in the older study the agreement was closer.

Substantial concordance with the Minder&Zingg study also emerged for the proportions of agreement between principal hospital diagnosis and combined primary and contributory cause of death information. The figures from our study show a closer agreement for several cancers (such as larynx, oropharynx and colon) and, most clearly, for chronic lower respiratory disease, however again less agreement in the case of diabetes mellitus. At least for cancers the proportions of agreement were also very similar as in a large Swedish study based on 1995 data [13]. This high degree of similarity of patterns also supports the notion that different evaluation of complex situations (see e.g. [32]) rather than insufficient reporting is the main reason for the variance between hospital discharge and cause of death registration.

In line with others [1, 6] disagreement increased with age. Our data support the notion that this may be related to an increasing number of reported diagnoses/causes [1].

Our data do not suggest substantial variation in the individual agreement between male and female decedents, which is in line with Alpérovitch et al. [6] who however could establish associations with determinants not available in our study (history of vascular diseases, presence of incapacities, MMSE <24, >5 medications).

The odds ratios resulting from multiple logistic regression (Table 3) however suggest substantial variation in reporting and maybe in diagnostic practice that can not entirely be explained by age and cause of death of the deceased. Comparatively lower agreement for the less educated and those speaking a non-European language could point to a lower standard of reporting (and maybe also examination) among less advantaged patients. These findings and the substantial regional variation indicate potential for improving procedures in cause of death as well as hospital discharge reporting.

What measures could improve the quality of the MS and the cause of death statistics in Switzerland? First, the responsible authority and data owner (Swiss Federal Statistical Office, SFSO) could do much more than at present. Currently, the SFSO publishes data reports on a yearly basis and researchers can request access to individual data, but there are no published reports about data quality. Second, the regular use of data automatically helps to improve data quality, because problems become visible and can be overcome. Especially studies combining information from different data sources may promote the evaluation of corresponding strengths and limits [33]. Third, a case review based on full clinical documentation should be carried out for the areas with lower scores in this study. For example, a sample of deaths due to diabetes could be studied in detail. The example of cancer shows that the work of cancer registries has an impact on medical documentation. An individual comparison of the cause of death statistics with the data of the cancer registries is required by law from 2020. Finally, the instruction in the scope and methods of the cause of death statistics during the basic and continuing education of physicians should be improved.

### Strengths and limitations

To our best knowledge, there are only three other studies conducted in a general population and with an equivalent sample size [13, 15, 17], two of them however analysing quite old data from 2005 [15] or even 1995 [13]. The equal consideration of both points of view, from underlying cause of death to hospital diagnoses and from principal hospital discharge diagnosis to multiple causes of death, allowing comparisons in both directions, is quite exceptional. An additional strength is the contribution of evidence whether in disease categories with not clearly distinguishable subcategories, the master or subcategories should be preferred. Compared to other studies reporting decreasing information on secondary causes of death for those aged over 85 [15], our data showed for those deceased under 95 no evidence of decreasing data quality with increasing age.

The study has several limitations. First, agreement rates show reproducibility rather than accuracy of information. Since both, causes of death and hospital diagnoses generally stem from the same source, they equally may be wrong [12]. High reported rates of diagnostic disagreement among medical referrals [32] call for prudence. Second, our study was restricted to individuals deceased in a hospital. Agreement between physician’s diagnosis and cause of death may be substantially higher among persons who died in a hospital than among those who died elsewhere [13]. Empirical evidence is however conflicting, with studies endorsing [34] or contradicting this hypothesis [6] or showing contradictory results depending on cause of death [15]. Third, we had to exclude external causes. These were, however – closely after cancer – the category with best agreement in an autopsy study [34].

## Conclusion

Depending on cause of death / hospital diagnosis, the patterns of agreement vary widely, but do not substantially differ from those found in other studies. This also and notably applies to interrater reliability studies as well as studies comparing individual data from different data sources, supporting the view that a substantial proportion of variation is due to different evaluation of complex situations by individual physicians.

The agreement rates in this study also did only slightly differ from those found almost 30 years ago, in spite of a necropsy study suggesting a significant decline of major discrepancies between 1972 and 1992 [35].

Agreement at the individual level remains limited and suggests a potential for improving data quality [14]. Even if this should not affect the reliability of population frequencies [10], the substantial regional variation (with lower agreement rates in French-speaking populations) hints at differential exploitation of available diagnosis information for cause of death statistics. Potentially worrying is the evidence for lower retrieval performance of the underlying cause of death in hospital diagnoses among socially disadvantaged groups like single, less educated, or culturally less integrated persons.

For all these reasons, studies of this kind should be regularly conducted as a quality monitoring of hospital diagnoses and causes of death.

## Abbreviations

AMI: Acute myocardial infarction
COPD: Chronic obstructive pulmonary disease
ICD: International Statistical Classification of Diseases and Related Health Problems
MMSE: Mini-Mental State Examination
MS: Medical Statistics of Swiss Hospitals
WHO: World Health Organization

## Footnotes

1 Minimal death count reached only regarding “definitive” cause of death: Malignant neoplasm of eye and adnexa; Obesity; Multiple sclerosis; Crohn’s disease and ulcerative colitis; Rheumatoid arthritis and other inflammatory polyarthropathies; Hyperplasia of prostate; Congenital malformations of the circulatory system.

2 Minimal death count reached only regarding principal hospital discharge diagnosis: Malnutrition; Volume depletion; Mood [affective] disorders; Neurotic, stress-related and somatoform disorders; Transient cerebral ischaemic attacks and related syndromes; Gastritis and duodenitis.
